# The association between vitamin B12, albuminuria and reduced kidney function: an observational cohort study

**DOI:** 10.1186/1471-2369-16-7

**Published:** 2015-02-02

**Authors:** Gearoid M McMahon, Shih-Jen Hwang, Rikki M Tanner, Paul F Jacques, Jacob Selhub, Paul Muntner, Caroline S Fox

**Affiliations:** National Heart, Lung and Blood Institute’s Framingham Heart Study and the Center for Population Studies, 73 Mt. Wayte Ave, Suite 2, Framingham, MA 01702 USA; Renal Division, Brigham and Women’s Hospital and Harvard Medical School, Boston, MA USA; Department of Epidemiology, University of Alabama at Birmingham, Birmingham, AL USA; USDA Human Nutrition Center on Aging, Tufts University, Boston, MA USA; Division of Endocrinology, Brigham and Women’s Hospital and Harvard Medical School, Boston, MA USA

**Keywords:** Homocysteine, Albuminuria, Vitamin B12, Reduced kidney function

## Abstract

**Background:**

Variants in *CUBN*, the gene encoding cubilin, a proximal tubular transport protein, have been associated with albuminuria and vitamin B12 (B12) deficiency. We hypothesized that low levels of B12 would be associated with albuminuria in a population-based cohort.

**Methods:**

We analyzed participants from the Framingham Heart Study (n = 2965, mean age 58 years, 53% female) who provided samples for plasma B12. Logistic regression models adjusted for covariates including homocysteine were constructed to test the association between B12 and prevalent albuminuria (UACR ≥17 mg/g [men] and ≥25 mg/g [women]) and reduced kidney function (defined as an eGFR < 60 ml/min/1.73 m^2^, RKF). Because of a significant interaction between B12 and homocysteine in the prevalent RKF model (p = 0.005), the model was stratified by the median homocysteine levels. Logistic regression models were constructed to test the association between B12 and incident albuminuria and RKF. The results were replicated in 4445 participants from NHANES 2003–2004.

**Results:**

Baseline B12 levels ranged from 50-1690 pg/ml. Elevated B12 was associated with prevalent albuminuria (OR 1.44 per 1 SD increase, 95% CI 1.10-1.87) and RKF (OR 1.83, 95% CI 1.30-2.60). However after stratifying by median homocysteine levels, this relationship remained only in the higher homocysteine stratum. There was no association between B12 and incident albuminuria (OR 1.17, 95% CI 0.79 – 1.73) or RKF (OR 1.45, 95% CI 0.97 – 1.88). In the NHANES cohort, elevated B12 was associated with RKF after full covariate adjustment (OR 3.06, 95% CI 2.30-4.08). There was no association with albuminuria.

**Conclusion:**

In participants with high baseline homocysteine levels, increased plasma B12 was associated with RKF.

## Background

Vitamin B12 (B12) is a water-soluble vitamin that plays a key role in the normal function of the nervous system and blood formation along with serving as a cofactor for the formation of methionine from homocysteine [1]. Normally, B12 is released from dietary protein in the stomach and binds to intrinsic factor (IF). The B12-IF complex is absorbed in the ileum via the cubilin receptor [1]. Defects in cubilin, a proximal tubular membrane protein, have been associated with both megaloblastic anemia and tubular proteinuria [1–4]. Cubilin also acts to reabsorb the majority of filtered albumin from the urine and recently, genome-wide association studies have identified SNPs in *CUBN* in association with albuminuria [5] and B12 levels [6, 7].

B12 deficiency is associated with anemia and neurological disorders. Through its central role in homocysteine metabolism, low B12 levels have been associated with higher serum homocysteine, a risk factor for cardiovascular disease [3]. However, in CKD homocysteine levels increase as GFR declines, independent of B12 levels [8].

Thus, there is a complex relationship between the processing of B12 and albumin in the gut and the kidney. The purpose of this study was to evaluate the association between B12 levels, albuminuria and reduced kidney function (RKF). We hypothesized that lower levels of B12 would be associated with an increased risk of albuminuria and RKF and that this association would be modified by homocysteine. To study this, we examined these associations in participants from the Framingham Heart Study. We further replicated our results in participants from the National Health and Nutrition Examination Survey (NHANES) 2003–2004. To account for the independent relationships of homocysteine with CKD and serum B12 levels, we further adjusted for baseline homocysteine levels.

## Methods

### Study samples

Framingham Heart Study (FHS) participants were drawn from the Offspring Study cohort [9]. Participants in the Offspring cohort are the children and spouses of the original Framingham cohort who underwent assessments in 4–8 year cycles including a physical examination, blood and urine biochemistries, assessment of cardiovascular risk factors and a physician interview. To be included in this study, participants were required to have attended the Offspring sixth examination cycle (1995–1998), provided a serum sample for B12 and provided a specimens to measure the urinary albumin/creatinine ratio (UACR) [albuminuria analysis] and serum creatinine [RKF analysis]. In total 3,532 participants attended the sixth examination cycle. Of these, 2965 participants provided samples for UACR and B12 measurement and were included in the albuminuria analysis. For the RKF analysis, 3,464 participants provided samples for serum creatinine and B12 measurements. Participants missing covariates (n = 13) were excluded leaving 3,451 participants in the final RKF analysis. All participants provided written informed consent. The study was approved by the Institutional Review Board at Boston University.

NHANES 2003–2004 was a stratified, multistage probability survey designed to select a representative sample of the civilian non-institutionalized US population. This examination cycle was chosen as it was the only one where both B12 and homocysteine were available. After excluding participants younger than 18 years of age and those participants lacking a measurement of B12, urinary albumin and creatinine or serum creatinine, 4,445 participants were included in the analysis of B12 and RKF and 4,395 participants were included in the analysis of B12 and albuminuria. The protocol for NHANES 2003–2004 was approved by the National Center for Health Statistics of the Center for Disease Control and Prevention Institutional Review Board. All participants gave informed consent.

### Measurement of B12

FHS participants provided fasting morning plasma samples that were stored at −80°C until processing. B12 was measured using a chemoluminescence assay (Immulite 1000 Immunoassay, Siemens). The coefficient of variation of the assay was 9.3%.

In NHANES 2003–2004, levels of B12 were measured using Bio-Rad Laboratories Quantaphase II Folate/B12 radioassay kit.

### Albuminuria

In FHS participants, UACR was measured on spot urine samples at the Offspring sixth and eighth examination cycles. Urine samples were collected and stored at −20°C. The urinary albumin concentration was assayed using immunoturbidimetry (Tina Quant Albumin Assay, Roche Diagnostics). Urinary creatine was measured using the modified Jaffé method (Colorimetric Assay, Roche Diagnostics). We defined albuminuria defined as a UACR ≥17 mg/g in men and ≥25 mg/g in women at exam 6. Incident albuminuria was defined as albuminuria at exam 8 in participants without albuminuria at exam 6.

In NHANES 2003–2004, urinary albumin was measured by solid phase fluorescence immunoassay and creatinine was measured by the modified kinetic Jaffe method on random spot urine samples.

### Reduced kidney function

Serum creatinine levels were assayed using the modified Jaffé method in both FHS and NHANES. We defined RKF as an estimated glomerular filtration rate (eGFR) <60 ml/min/1.73 m^2^ using the CKD-EPI equation [10]. Incident RKF was defined as the development of RKF by the Offspring eighth examination cycle (2005–2008) in FHS participants.

### Covariate assessment

Covariates were obtained from FHS participants at the baseline examination. Systolic and diastolic blood pressures were recorded as the average of two physician-performed measurements. Hypertension was defined as a systolic blood pressure of ≥140 mmHg, a diastolic blood pressure of ≥90 mmHg or the use of antihypertensive medications. Serum glucose and high density lipoprotein cholesterol (HDL_c_) were measured on fasting blood samples. Diabetes mellitus was defined as a fasting blood sugar of ≥126 mg/dl or treatment with a hypoglycemic agent. Dipstick proteinuria was defined as the presence of trace or more proteinuria on dipstick (Ames Labstix, Elkhart, Indiana). Participants were considered current smokers if they had smoked ≥1 cigarette per day over during the previous year. The body mass index (BMI) was defined as the participant’s weight in kilograms divided by their height in meters squared. Plasma homocysteine was measured by high-performance liquid chromatography with fluorimetric detection. The coefficient of variation of the assay was 9%.

In NHANES 2003–2004, questionnaires were used to assess participants’ age, sex, smoking status, prior diagnosis of hypertension and diabetes and use of antihypertensive and hypoglycemic medications. Blood pressure was measured three times by a trained physician following a standardized protocol. Hypertension was defined as systolic blood pressure ≥ 140 mm Hg, diastolic blood pressure ≥ 90 mm Hg, or use of antihypertensive medication. Serum glucose was measured using a Beckman Synchron LX20 analyzer and diabetes was defined as a fasting glucose ≥ 126 mg/dL, non-fasting glucose ≥ 200 mg/dL, or a self-reported history of diabetes with concurrent use of hypoglycemic medication. HDL_c_ was measured using the Hitachi 704 Analyzer. Plasma homocysteine was measured using a fluorescence polarization immunoassay (Abbott Diagnostics, Lake Forest, IL).

### Statistical analysis

We grouped the baseline characteristics of the FHS participants by quartile of B12 level. We calculated the statistical significance of differences using χ^2^ test for categorical variables and ANOVA for continuous variables. Because the B12, homocysteine and UACR were not normally distributed, these were log-transformed for the purpose of the analysis. The association between B12 and eGFR, systolic blood pressure, HDL_c_, BMI, UACR and homocysteine was estimated using Pearson correlation coefficients adjusted for age and sex.

We constructed a logistic regression model, adjusted for age and sex, to test the association between B12 and albuminuria. Log-transformed B12 was treated as a continuous variable and odds ratios (OR) were calculated per 1 standard deviation increase. We further adjusted the model for systolic blood pressure, HDL_c_, smoking, hypertension treatment and the presence of diabetes. Because elevated homocysteine is associated with CKD and albuminuria and homocysteine levels are known to be altered by B12 [3], the final model included an adjustment for baseline homocysteine levels. We then repeated these analyses after stratifying participants by the median baseline homocysteine levels.

We tested the relationship between B12 and RKF at the baseline examination using a logistic regression model adjusted for age, sex, diabetes, hypertension and dipstick proteinuria. The model was further adjusted for baseline homocysteine levels. Because homocysteine significantly modified the relationship between B12 and RKF, we repeated the analysis after stratifying by the median baseline homocysteine levels.

We used a logistic regression model to estimate the association between B12 and incident albuminuria in FHS participants, defined as UACR ≥17 mg/g in men and ≥25 mg/g in women at Offspring examination cycle 8 [2005–2008]. Participants with albuminuria at the baseline examination were excluded. The incident albuminuria model was adjusted for age, sex, systolic blood pressure, HDL_c_, smoking status, hypertension treatment and the presence of diabetes. We then further adjusted the model for baseline homocysteine levels. Because there was no interaction between B12 and homocysteine in the incident analyses, no stratified analysis was done.

We tested the association between B12 and incident RKF using a logistic regression model. The multivariable adjusted model included age, sex, baseline eGFR, diabetes, hypertension and dipstick proteinuria. This model was again adjusted for baseline homocysteine levels. Statistical analyses were performed using SAS, version 9.2 (Cary, North Carolina). A two-tailed p-value of <0.05 was considered significant.

To replicate our findings, we constructed identical logistic regression models to test the cross-sectional association between B12, albuminuria and RKF in participants from NHANES 2003–2004. The models were adjusted and stratified as described above for FHS. Analyses for NHANES 2003–2004 were performed using SUDAAN 10.1 (Research Triangle Institute, Research Triangle Park, NC) accounting for this study’s complex sampling design, including unequal probabilities of selection, over-sampling, and non-response. Sampling weights were applied for all NHANES 2003–2004 analyses to produce estimates that are representative of the national U.S population.

## Results

### Baseline characteristics

In total, 2,965 FHS participants were included in the study. Baseline B12 ranged from 50–1690 pg/ml. In total, 135 participants had B12 levels below 200 pg/ml. The characteristics of the participants, stratified by quartile of vitamin B12, are shown in Table 1. There was no difference in baseline eGFR, UACR or the prevalence of albuminuria across vitamin B12 quartiles. There was an inverse relationship between vitamin B12 and homocysteine levels across quartiles (p < 0.0001 for trend).

**Table 1:** **Baseline characteristics of the Framingham Offspring Study participants stratified by quartiles of serum B12**

	Quartile 1	Quartile 2	Quartile 3	Quartile 4	
Characteristic	Mean/n/median	Mean/n/median	Mean/n/median	Mean/n/median	p-value*
N	741	741	742	741	
B12, mean, pg/ml (SD)	243 (47)	348 (25)	444 (32)	648 (153)	
B12, range, pg/ml (IQR)	50-306	307-393	394-502	503-1690	
Age, years (SD)	58.7 (10.0)	58.4 (9.9)	58.5 (9.4)	58.7 (9.6)	0.92
Sex, n (%women)	382 (51.6)	359 (48.4)	379 (51.1)	458 (61.8)	0.16
Diabetes, n, (%)	64 (8.6)	63 (8.5)	81(10.9)	73 (9.8)	0.07
Systolic BP, mmHg (SD)	128.1 (18.5)	128.8 (19.5)	128.8 (18.4)	128.1 (19.1)	0.81
Diastolic BP, mmHg (SD)	75.6 (9.3)	75.7 (9.9)	75.7 (9.3)	78.1 (9.3)	0.81
Hypertension Treatment, n (%)	207 (28.0)	207 (27.9)	206 (27.7)	199 (26.9)	0.98
Body Mass Index, kg/m^2^ (SD)	28.3 (5.0)	28.0 (4.9)	28.2 (5.7)	27.2 (5.0)	<0.001
HDL_c_, mg/dl (SD)	50.2 (15.4)	50.0 (16.1)	51.1 (16.1)	52.6 (16.9)	0.17
Current Smoker, n (%)	116 (15.7)	119 (16.1)	112 (15.1)	103 (13.9)	0.51
eGFR, ml/min/1.73 m^2^ (SD)	85.1 (18.2)	85.3 (19.1)	84.6 (18.6)	84.9 (19.5)	0.68
RKF, n (%)	87 (10.1)	72 (8.4)	78 (9.0)	82 (9.5)	0.91
UACR, mg/g (IQR)	6.1 (2.7-13.5)	5.9 (2.5-15.6)	6.1 (2.9-14.9)	7.6 (3.2-17.4)	0.49
Microalbuminuria, n (%)	115 (15.5)	133 (17.9)	131 (17.7)	144 (19.4)	0.1
Homocysteine, μmol/L (SD)	11.2 (5.1)	9.9 (3.5)	9.2 (2.8)	8.6 (3.0)	<0.001

*Test for significance of linear trend adjusted for age and sex.

Plasma B12 was weakly correlated with HDL_c_ (r = 0.06, p = 0.002) and BMI (r = −0.08, p < 0.001). There was an expected negative correlation with plasma homocysteine levels (r = −0.29, p < 0.001). There was no correlation between B12 levels and UACR or eGFR.

### Cross-sectional analyses of B12 with albuminuria

In the cross-sectional age- and sex-adjusted and multivariable-adjusted models, there was no association between levels of B12 and prevalent albuminuria (Table 2) in FHS participants. However, after adjustment for homocysteine levels, higher B12 levels were associated with an increased risk of albuminuria (OR 1.44 per 1 SD increase, 95% CI 1.10 – 1.87). There was no interaction between B12 and homocysteine (p_interaction_ = 0.63). When the participants were stratified by homocysteine levels, there was no association between B12 and albuminuria after full covariate adjustment (OR 1.27, 95% CI 0.85 – 1.90) in participants with homocysteine levels < 9.08 μmol/L. However, in participants with homocysteine above the median, higher B12 levels were associated with albuminuria (OR 1.57, 95% CI 1.10 – 2.26).

**Table 2:** **The cross-sectional association between B12 (OR per 1SD increase log Vitamin B12) and prevalent albuminuria and RKF at Offspring Examination 6**

			Model 1			Model 2			Model 3		
	n	Events (%)	OR	CI	p-value	OR	CI	p-value	OR	CI	p-value
Entire Cohort											
Albuminuria	2965	523 (17.6)	1.25	0.98-1.60	0.07	1.25	0.97-1.61	0.08	1.44	1.10-1.87	0.008
RKF	3451	319 (9.2)	0.99	0.72-1.35	0.94	0.98	0.72-1.35	0.92	1.83	1.30-2.59	<0.001
Stratified analysis: Albuminuria*											
Homocysteine < median	1511	228 (15.1)	1.28	0.87-1.88	0.21	1.29	0.87-1.91	0.21	1.27	0.85-1.90	0.24
Homocysteine ≥ median	1454	295 (20.3)^†^	1.45	1.03-2.03	0.03	1.41	0.99-2.01	0.06	1.57	1.10-2.26	0.01
Stratified analysis: RKF*											
Homocysteine < median	1722	70 (4.1)	0.90	0.47-1.75	0.76	0.92	0.47-1.78	0.80	1.22	0.62-2.41	0.56
Homocysteine ≥ median	1729	249 (14.4)^†^	1.62	1.10-2.37	0.01	1.57	1.06-2.31	0.02	2.17	1.44-3.26	<0.001

Model 1: Age- and sex- adjusted.

Model 2: Multivariable-adjusted: Albuminuria - systolic blood pressure, HDL_c_, smoking status, hypertension treatment and diabetes.

RKF: diabetes, hypertension and dipstick proteinuria.

Model 3: Multivariable-adjusted + baseline log homocysteine.

*Stratified by the median homocysteine levels (9.08 μmol/L).

^†^p-value <0.001 for comparison.

### Cross-sectional analyses of B12 with RKF

In cross-sectional age- and sex-adjusted models and the multivariable model, there was no association between B12 and RKF (Table 2). After adjusting for homocysteine, there was an association between B12 and RKF (OR 1.83, 95% CI 1.30 – 2.60). The association between B12 and RKF differed by level of homocysteine (p_interaction_ = 0.005). After stratifying by median homocysteine (9.08 μmol/L), the relationship between B12 and RKF remained robust in participants with homocysteine ≥ 9.08 μmol/L (OR 2.17, 95% CI 1.44 – 3.26). However, at lower levels of homocysteine, there was no association between B12 and RKF (OR 1.22, 95% CI 0.62 – 2.41).

### B12 in association with incident albuminuria

Of those patients (n = 1931) who returned for the follow-up examination and provided samples for UACR, 214 (11.1%) developed albuminuria. There was no association between B12 levels and incident albuminuria after multivariable adjustment (OR 0.96, 95% CI 0.66-1.39, Table 3) or after further adjustment for baseline homocysteine levels (OR 1.17, 95% CI 0.79 – 1.73, Table 3). There was no interaction between B12 and homocysteine (p = 0.11).

**Table 3:** **The association between B12 and incident albuminuria and incident RKF in the Framingham Heart Study**

			Model 1			Model 2			Model 3		
	n	Events (%)	OR	CI	p-value	OR	CI	p-value	OR	CI	p-value
Albuminuria	1931	214 (11.1)	0.96	0.67-1.38	0.84	0.96	0.66-1.39	0.81	1.17	0.79-1.73	0.44
RKF	2145	237 (11.0)	1.00	0.72-1.53	0.81	0.99	0.69-1.53	0.90	1.32	0.87-2.02	0.19

Model 1: Age- and sex- adjusted.

Model 2: Multivariable-adjusted: Albuminuria - systolic blood pressure, HDL_c_, smoking status, hypertension treatment and diabetes.

RKF: diabetes, hypertension, baseline eGFR and dipstick proteinuria.

Model 3: Multivariable-adjusted + baseline lnhomocysteine.

OR are presented per 1SD increase log Vitamin B12.

### B12 in association with incident RKF

For the analysis of the association between B12 and incident RKF, 2,382 participants were included (Table 3). In total, 237 participants had an RKF at the follow-up examination (11.0%). There was no association between B12 and incident RKF after multivariable adjustment either before (OR 0.99, 95% CI 0.69-1.53) or after baseline homocysteine adjustment (OR 1.32, 95% CI 0.87 – 2.02). Finally, there was no interaction between B12 and homocysteine (p = 0.06).

### External replication in NHANES 2003–2004 participants

In contrast to the results in FHS participants, in the NHANES cohort, there was no association between B12 and albuminuria in the unadjusted and multivariable-adjusted analysis or after adjustment for homocysteine levels (Table 4). No association was present between vitamin B12 and albuminuria for NHANES participants with homocysteine levels above or below the median (8.34 μmol/L).

**Table 4:** **The cross-sectional association between B12 (OR per 1SD increase lnB12) and prevalent albuminuria and RKF in the NHANES 2003–2004 study**

			Model 1			Model 2			Model 3		
	n	Events (%) ^1^	OR	CI	p-value	OR	CI	p-value	OR	CI	p-value
Entire cohort											
Albuminuria	4395	764 (13.3)	1.05	0.87-1.27	0.56	1.13	0.88-1.45	0.26	1.16	0.89-1.50	0.26
RKF	4445	489 (7.0)	1.13	0.85-1.51	0.37	1.17	0.85-1.61	0.32	3.06	2.30-4.08	<0.001
Stratified analysis: Albuminuria^2^											
Homocysteine < median	2196	277 (11.5)	1.14	0.86-1.51	0.32	1.27	0.88-1.83	0.18	1.24	0.84-1.83	0.26
Homocysteine ≥ median	2197	487 (15.1)	1.05	0.80-1.36	0.72	1.05	0.79-1.40	0.26	1.18	0.88-1.59	0.26
Stratified analysis: RKF^2^											
Homocysteine < median	2222	18 (0.7)	0.64	0.15-2.74	0.52	0.63	0.14-2.85	0.52	0.62	0.14-2.76	0.51
Homocysteine ≥ median	2223	471 (13.3)	1.56	1.16-2.09	0.01	1.59	1.16-2.17	0.01	3.46	2.64-4.53	<0.001

Model 1: Age- and sex- adjusted.

Model 2: Multivariable-adjusted: Albuminuria - systolic blood pressure, HDL_c_, smoking status, hypertension treatment and diabetes.

RKF: diabetes, hypertension and dipstick proteinuria.

Model 3: Multivariable-adjusted + lnhomocysteine.

^1^Percentages are weighted to reflect the US population and take into account the multi-stage complex sampling design used to enroll NHANES participants.

^2^Stratified by the median homocysteine levels (8.34 μmol/L).

Similar to the FHS cohort, there was no cross-sectional association between vitamin B12 and RKF in the age- and sex-adjusted and multivariable-adjusted models. After further adjustment for homocysteine levels, there was an association between B12 and RKF (OR 3.06, 95% CI 2.30-4.08, Table 4). After stratifying by the median homocysteine level, there was an association between B12 and RKF in the higher homocysteine stratum (OR 3.46, 95% CI 2.64-4.53) but not in the lower homocysteine stratum (OR 0.62 95% CI 0.14-2.76). There was a significant interaction between B12 and homocysteine (p_interaction_ <0.001).

## Discussion

The findings of this study are fourfold. First, we found that B12 was not associated with albuminuria or RKF in the univariate or multivariable-adjusted model. However, elevated levels of B12 were associated cross-sectionally with a higher odds of albuminuria after adjusting for the plasma homocysteine concentration. After further stratification by the median homocysteine concentration, the association between B12 and albuminuria remained only in the higher homocysteine group. However, this result did not replicate in the NHANES cohort.

Third, elevated B12 levels were associated cross-sectionally with a higher odds of RKF after adjusting for the plasma homocysteine concentration. The association between B12 and RKF remained only in the higher homocysteine group after stratification by the median homocysteine level. This result was confirmed in participants from the NHANES cohort. Finally, there was no association between B12 levels and incident albuminuria and RKF.

### In the context of the current literature

B12 levels are a function of dietary intake and deficiencies result from reduced intake or decreased absorption in the ileum. Defects in gut and proximal tubular transport proteins have been associated with albuminuria and B12 deficiency [1, 4]. B12 deficiency has been well described but elevations in B12 levels have not been as well studied. There is no upper recommended daily limit for B12 as there are no documented cases of toxicity [11]. However, elevations in B12 have been noted in association with a variety of conditions including liver disease, malignancies, and inflammatory disorders [12]. The strongest predictor of elevated B12 levels in hospitalized patients is CKD [13, 14]. The mechanism for this increase is unclear. Under normal circumstances, although B12 is filtered at the glomerulus, excretion in the urine is minimal due to reabsorption in the proximal tubule [3]. As such, high B12 levels found in the setting of CKD should not be related to decreased clearance. However, in the setting of exogenous administration of B12, the kidneys are an important route for excretion as the reabsorptive mechanism is saturable. Thus, the administration of supraphysiologic doses of B12 to individuals with CKD could lead to an elevated in the serum concentration.

Typically there is an inverse relationship between levels of homocysteine and B12. However, this relationship is altered in patients with CKD. Although B12 supplementation reduces homocysteine levels in patients with CKD, it is less effective than in patients with normal renal function [5]. In patients on hemodialysis, continuous replacement of B12 is required to maintain lower homocysteine levels despite normal plasma B12 values [15].

In this study, as expected, we found that B12 was inversely associated with homocysteine. B12 levels were not associated with RKF prior to adjustment for homocysteine or in individuals with homocysteine levels below the median. This suggests that elevated B12 alone is not associated with an increased risk for RKF. However, in patients with elevated homocysteine levels, higher vitamin B12 concentrations were associated with an increased prevalence of RKF. The combination of elevated homocysteine along with increased B12 suggests the possibility of a resistance to the usual effects of B12 in these individuals.

### Potential mechanisms

B12 in the blood is primarily protein-bound. Approximately 20% of circulating B12 is bound to holotranscobalamin (TC2) with the remainder to haptocorrin [16]. TC2-bound B12 is the biologically active form as B12 bound to haptocorrin cannot be taken up into cells [16]. A congenital form of megaloblastic anemia has been described in infants lacking TC2 despite normal total B12 levels [17]. The kidney plays an important role in TC2 metabolism. TC2 is filtered at the glomerulus and is reabsorbed in the proximal tubule by megalin. B12 is then returned to the blood bound to newly synthesized TC2 [18]. Thus, defects in protein reabsorption in the proximal tubule could lead to a loss of biologically active TC2 in the urine.

Increased TC2 and haptocorrin levels have been noted in patients with CKD [13]. Despite this, there is decreased uptake of TC2 into cells [18, 19]. This can lead to a paradoxical increase in cellular homocysteine levels despite normal total B12. Thus, a functional B12 deficiency can occur in patients with CKD in the setting of increased TC2 losses in the urine, decreased TC2 absorption in the proximal tubule and decreased cellular uptake of TC2.

Another important consideration is the possibility that elevated B12 levels and B12 supplementation may be harmful in individuals with CKD. Cyanide metabolism is abnormal in individuals with CKD due to decreased clearance [20]. Cyanocobalamin, the most commonly used form of B12 replacement is metabolized to active methylcobalamin releasing small amounts of cyanide. Under normal circumstances, methylcobalamin acts as a means of removing cyanide from the circulation through conversion to cyanocobalamin. However, in patients with CKD the reduced cyanide clearance prevents conversion of cyanocobalamin to the active form and therefore supplementation in this form is less effective at reducing homocysteine levels [21].

In fact, a recent randomized trial of cyanocobalamin administration found more rapid GFR decline and cardiovascular morbidity in the treatment arm [22]. It was suggested that this may be related to increased synthesis of assymetric dimethylarginine which is known to inhibit nitric oxide. However, the data above suggest that in fact this finding may result from the use of cyanocobalamin rather than methylcobalamin in this population and that methylcobalamin should be preferred in individuals with CKD. Unfortunately, as we do not have data on vitamin supplementation, this is speculative and would warrant further investigation.

### Implications

There are a number of implications to this study. First, although previous studies have demonstrated that total B12 levels may not accurately reflect B12 status in patients with CKD, our study shows that this is true even in individuals with moderate declines in renal function. This, however, is complicated by the general increase in homocysteine seen in patients with CKD that is unrelated to B12 levels [12]. The appropriate range of B12 levels in CKD remains to be defined adequately. Downstream metabolites such as methylmalonic acid and homocysteine may more accurately reflect functional B12 status in patients with CKD. Similarly, whether monitoring TC2 levels rather than total B12 is more appropriate in patients with CKD is unclear and warrants further study. The means of B12 supplementation chosen may be important in individuals with CKD.

Finally, although B12 was associated cross-sectionally with RKF, there was no association with incident RKF or albuminuria. This suggests that the altered metabolism of B12 is present only in individuals with established RKF and that elevated levels are not a risk factor for future CKD. This is supported by the fact that B12 supplementation was found to be harmful only in individuals with GFR < 50 ml/min/1.73 m^2^ [22] and had no effects (positive or negative) in subjects with normal renal function. All of the patients in the incident RKF analysis had an eGFR >60 ml/min/1.73 m^2^ at the time that B12 and homocysteine were measured.

### Strengths and limitations

The well-characterized Framingham Heart Study and well-defined cardiovascular risk factors are an important strength of this study. We performed independent replication of our findings in the NHANES study. However, there are a number of limitations that should be stated. B12 levels are related to dietary intake and the use of supplements which were not recorded [23, 24]. Similarly, the influence of folate supplementation on homocysteine levels is substantial and may introduce some bias by altering the relationship between B12 and homocysteine. Albuminuria and RKF were defined on the basis of single measurements which might lead to some misclassification. Our findings are observational and causality cannot be inferred.

## Conclusion

In subjects with high homocysteine levels, increased plasma B12 was associated with prevalent RKF.

## Abbreviations

CKD: Chronic kidney disease
RKF: Reduced kidney function
B12: Vitamin B12
IF: Intrinsic factor
NHANES: National health and nutrition examination study
FHS: Framingham heart study
UACR: Urinary albumin/Creatinine ratio
HDL_c_: High density lipoprotein cholesterol
BMI: Body mass index
OR: Odds ratio
TC2: Holotranscobalamin
eGFR: Estimated glomerular filtration rate.
